# Safety of bazedoxifene in a randomized, double-blind, placebo- and active-controlled phase 3 study of postmenopausal women with osteoporosis

**DOI:** 10.1186/1471-2474-11-130

**Published:** 2010-06-22

**Authors:** Claus Christiansen, Charles H Chesnut, Jonathan D Adachi, Jacques P Brown, César E Fernandes, Annie WC Kung, Santiago Palacios, Amy B Levine, Arkadi A Chines, Ginger D Constantine

**Affiliations:** 1 Center for Clinical and Basic Research, Ballerup, Denmark; 2 University of Washington, Seattle, Washington, USA; 3 St. Joseph's Healthcare, McMaster University, Hamilton, Ontario, Canada; 4 CHUL Research Centre, Laval University, Québec City, Québec, Canada; 5 ABC Medical School, São Paulo, Brazil; 6 The University of Hong Kong, Queen Mary Hospital, Hong Kong, China; 7 Instituto Palacios, Salud y Medicina de la Mujer, Madrid, Spain; 8 Pfizer Inc, Collegeville, Pennsylvania, USA

## Abstract

**Background:**

We report the safety findings from a 3-year phase 3 study (NCT00205777) of bazedoxifene, a novel selective estrogen receptor modulator under development for the prevention and treatment of postmenopausal osteoporosis.

**Methods:**

Healthy postmenopausal osteoporotic women (N = 7,492; mean age, 66.4 years) were randomized to daily doses of bazedoxifene 20 or 40 mg, raloxifene 60 mg, or placebo for 3 years. Safety and tolerability were assessed by adverse event (AE) reporting and routine physical, gynecologic, and breast examination.

**Results:**

Overall, the incidence of AEs, serious AEs, and discontinuations due to AEs in the bazedoxifene groups was not different from that seen in the placebo group. The incidence of hot flushes and leg cramps was higher with bazedoxifene or raloxifene compared with placebo. The rates of cardiac disorders and cerebrovascular events were low and evenly distributed among groups. Venous thromboembolic events, primarily deep vein thromboses, were more frequently reported in the active treatment groups compared with the placebo group; rates were similar with bazedoxifene and raloxifene. Bazedoxifene showed a neutral effect on the breast and an excellent endometrial safety profile. The incidence of fibrocystic breast disease was lower with bazedoxifene 20 and 40 mg versus raloxifene or placebo. Reductions in total and low-density lipoprotein levels and increases in high-density lipoprotein levels were seen with bazedoxifene versus placebo; similar results were seen with raloxifene. Triglyceride levels were similar among groups.

**Conclusion:**

Bazedoxifene showed a favorable safety and tolerability profile in women with postmenopausal osteoporosis.

**Trial Registration:**

**Trial registration number**: NCT00205777; **Trial registration date**: September 16, 2005

## Background

Postmenopausal women experiencing declining levels of endogenous estrogens are disproportionately affected by osteoporosis, which affects one third of women between the ages of 60 and 70 years and two thirds of women 80 years and older [1]. Numerous agents for the prevention and/or treatment of postmenopausal osteoporosis are currently available, including bisphosphonates, estrogen therapy, parathyroid hormone (PTH), calcitonin, and the selective estrogen receptor modulator (SERM) raloxifene [2]. Although existing pharmacologic agents for postmenopausal osteoporosis have been shown to be effective in preventing bone loss and fractures [3], there remains an ongoing need to provide women with new therapeutic options, primarily due to variable safety and tolerability profiles.

SERMs, also referred to as estrogen agonists/antagonists, are a structurally diverse group of compounds that can confer estrogen receptor agonist or antagonist effects depending on the target tissue, making them attractive candidates for the prevention and/or treatment of postmenopausal osteoporosis [4]. Bazedoxifene is a novel SERM that was developed using a stringent preclinical screening process designed to select compounds with favorable effects on bone and lipid profiles while minimizing stimulation of uterine or breast tissue [4,5]. In a phase 2 study of healthy postmenopausal women, daily oral doses of bazedoxifene 2.5, 5.0, 10, 20, 30, or 40 mg were generally well tolerated and did not stimulate the endometrium [6]. Moreover, bazedoxifene 30 and 40 mg showed significantly smaller increases in endometrial thickness and significantly reduced the incidence of uterine bleeding compared with placebo [6]. In a 2-year phase 3 study of postmenopausal women at risk for osteoporosis, bazedoxifene 10, 20, and 40 mg was shown to prevent bone loss and reduce bone turnover and was associated with a favorable endometrial, ovarian, and breast safety profile [7,8].

A pivotal, global phase 3 study in postmenopausal women with osteoporosis showed that bazedoxifene 20 and 40 mg and raloxifene 60 mg significantly reduced the risk of new vertebral fracture relative to placebo over 3 years of therapy [9]. A post hoc analysis of a subgroup of women at higher risk for fracture showed that bazedoxifene 20 mg significantly reduced the risk of nonvertebral fracture [9]. Here we report the results of the safety evaluations from that study.

## Methods

### Study design

Full details of the study design and methodology have been previously reported [9]. This was a 3-year multicenter, randomized, double-blind, placebo- and active-controlled phase 3 trial (NCT00205777) conducted at 206 sites worldwide (and continuing as an ongoing study extension for 4 additional years). Eligible subjects were generally healthy postmenopausal women aged 55 to 85 years with osteoporosis, defined as low bone mineral density (BMD) or radiographically confirmed vertebral fractures. Subjects without prevalent vertebral fracture were required to have lumbar spine or femoral neck BMD T-scores between -2.5 and -4.0 (inclusive), whereas subjects with prevalent vertebral fracture (at least 1 mild vertebral fracture) were required to have lumbar spine and femoral neck BMD T-scores not worse than -4.0.

Exclusion criteria included diseases that may affect bone metabolism or conditions that could interfere with measurement of BMD, pathologic vertebral fractures, vasomotor symptoms requiring treatment, active or past history of venous thromboembolic events (VTEs), endometrial hyperplasia or carcinoma, abnormal vaginal bleeding, or malignancy within 10 years of the study. Subjects were prohibited from the use of any drug known to have an effect on bone metabolism, including androgens, systemic estrogens (except estriol ≤2.0 mg/day), topical estrogens (>3 times/week), progestogens, SERMs, bisphosphonates, calcitonin, PTH, and cholecalciferol (>50,000 IU/week) within 6 months of screening.

Eligible subjects were randomly assigned to receive a once-daily oral dose of bazedoxifene 20 or 40 mg, raloxifene 60 mg, or placebo for 3 years. All subjects were to receive daily supplementation with oral calcium (1,000-1,200 mg) and vitamin D (400-800 IU).

Non-hysterectomized women were eligible for participation in an endometrial safety substudy designed to examine measures related to the endometrium and ovaries [10]. Subjects with endometrial thickness >5 mm as measured by transvaginal ultrasound (TVU) or endometrial hyperplasia or carcinoma on baseline biopsy were excluded from the substudy.

The study protocol and an informed consent form were submitted to the independent ethics committee or institutional review board at each institution for review and written approval. All subjects provided written informed consent prior to enrollment in the study, which was conducted in accordance with the ethical principles outlined in the Declaration of Helsinki.

### Assessments

Full details of the efficacy assessments and related findings have been reported elsewhere [9]. Subjects were monitored throughout the 3-year study, with clinic visits occurring quarterly during the first year and biannually during the second and third years.

Safety was monitored by means of physical examinations, gynecologic and breast examinations, mammography, cervical cytology smears, clinical laboratory determinations (including blood lipid assessments), and recording of adverse events (AEs), which were classified using the US Food and Drug Administration's Coding Symbols for Thesaurus of Adverse Reaction Terms (COSTART). For subjects enrolled in the endometrial safety substudy, TVU of the uterus and ovaries was performed at baseline and at Months 12 and 24 (or upon withdrawal from the study if more than 9 months had elapsed since the last assessment) and endometrial biopsies were performed at baseline and at Month 24. Mammography was performed at baseline and at Months 12, 24, and 36 (or upon withdrawal from the study if more than 9 months had elapsed since the last assessment). Subjects in the overall safety population who reported abnormal uterine bleeding at any time during the study were also evaluated by TVU and endometrial biopsy according to a protocol-specific algorithm for the assessment of uterine bleeding.

Independent blinded adjudication boards were formed to ensure a consistent, accurate, and unbiased assessment of the following AEs of interest: VTEs, stroke, transient ischemic attack (TIA), and breast cancer. The adjudication boards were composed of consultant physicians specializing in the fields of internal medicine with expertise in VTEs, cardiology, breast cancer, neurology, and neuroradiology. All adjudication board members reviewed each case independently in a blinded manner. Thereafter, a final decision was determined for each case based on majority or consensus decision, according to the guidance set forth in each of the adjudication board charters.

### Statistical Analyses

Safety data were analyzed in all subjects who received at least 1 dose of study medication. Differences in the incidence of AEs, serious AEs, and discontinuations due to AEs among treatment groups were evaluated using Chi-square analysis, with a significance level set at 0.05. The incidence of VTEs and cerebrovascular events, based on adjudicated data, was expressed as the rate in 1,000 women-years. Hazard ratios (HRs) of treatment versus comparator were calculated using a proportional hazard model without adjustment for possible covariates; corresponding 95% confidence intervals (CIs) and *P *values were obtained.

The median percent changes from baseline in lipid parameters at 36 months were analyzed using an analysis of covariance (ANCOVA) on ranked data, with treatment as factor and baseline as covariate.

## Results

### Subjects

**Figure 1:**
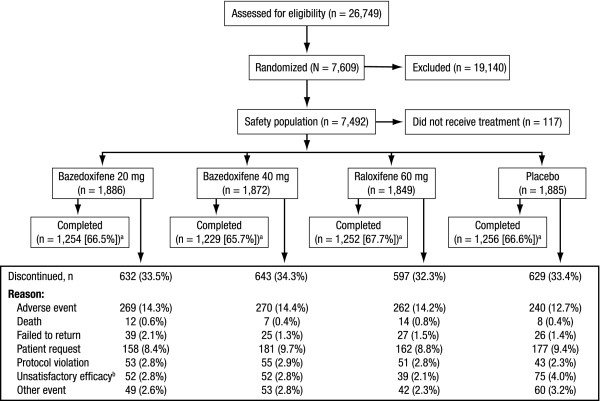
**Subject disposition**. The number of subjects who were screened, randomized and received at least 1 dose of study drug (safety population), and completed the study is shown. ^a^Does not include subjects (n = 92) who completed the 3-year core study and did not enter the extension study. ^b^Defined as occurrence of new vertebral fracture or ≥7% decrease in BMD of the lumbar spine or hip at any time during the study (*P *< 0.01 among treatment groups, Chi-square test).

Of 26,749 women screened for the study, 7,609 women were randomized to treatment with bazedoxifene 20 or 40 mg, raloxifene 60 mg, or placebo (Figure 1). A total of 7,492 women received ≥1 dose of study medication and were included in the safety population. Baseline demographic and clinical characteristics were generally similar among treatment groups (Table 1). The mean age of subjects was 66.4 years. The vast majority of women (91.2%) had experienced natural menopause, and the mean time since last menstrual period was 19.5 years.

**Table 1:** Baseline Characteristics (Safety Population)

	Bazedoxifene		Raloxifene	Placebo
Characteristic	20 mg (n = 1886)	40 mg (n = 1872)	60 mg (n = 1849)	(n = 1885)
Age, y				
Mean (SD)	66.5 (6.5)	66.2 (6.8)	66.4 (6.7)	66.5 (6.8)
Ethnic origin, n (%)				
White	1657 (87.9)	1623 (86.7)	1618 (87.5)	1641 (87.1)
Black	115 (6.1)	135 (7.2)	116 (6.3)	120 (6.4)
Hispanic	90 (4.8)	83 (4.4)	87 (4.7)	88 (4.7)
Other^a^	24 (1.3)	31 (1.7)	28 (1.5)	36 (1.9)
Years since last menstrual period				
Mean (SD)	19.7 (8.6)	19.3 (8.9)	19.5 (8.7)	19.5 (8.8)
Hysterectomy, n (%)	430 (22.8)	403 (21.5)	374 (20.2)	360 (19.1)
Type of menopause, n (%)				
Natural	1706 (90.5)	1690 (90.3)	1700 (91.9)	1738 (92.2)
Surgical oophorectomy	180 (9.5)	182 (9.7)	149 (8.1)	147 (7.8)
Subjects reporting hot flushes, n (%)^b^	287 (15.2)	316 (16.9)	264 (14.3)	251 (13.3)
Hot flushes per day				
Mean (SD)	2.2 (2.4)	2.1 (2.2)	1.9 (1.9)	2.0 (2.1)
BMI, kg/m^2^				
Mean (SD)	26.6 (3.8)	26.5 (3.9)	26.4 (3.8)	26.3 (3.8)

SD, standard deviation; BMI, body mass index.

^a^Includes Asian, Native American, Pacific Islander, and other ethnic origins.

^b^*P *< 0.05; Chi-square test.

There were no significant differences among treatment groups in the number of subjects who discontinued from the study (n = 2,501 [33%]; Figure 1). The most common reason for discontinuation was AEs. Overall, the incidence of AEs, serious AEs, and discontinuations due to AEs in the bazedoxifene treatment groups was not different from that seen in the placebo group (Table 2). Sixty-three subjects had deaths that resulted from AEs that began during the study and were reported to the sponsor: bazedoxifene 20 mg, n = 19 (1.0%); bazedoxifene 40 mg, n = 14 (0.7%); raloxifene 60 mg, n = 19 (1.0%); and placebo, n = 11 (0.6%). Differences between groups were not statistically significant.

**Table 2:** Overall Summary of AEs

	Bazedoxifene		Raloxifene	Placebo
Subjects, n (%)	20 mg (n = 1886)	40 mg (n = 1872)	60 mg (n = 1849)	(n = 1885)
Any AE	1807 (95.8)	1792 (95.7)	1777 (96.1)	1814 (96.2)
Any serious AE	382 (20.3)	368 (19.7)	344 (18.6)	354 (18.8)
Discontinuations due to AE	278 (14.7)	280 (15.0)	273 (14.8)	253 (13.4)
AEs > 10% in any treatment group				
Back pain	607 (32.2)	591 (31.6)	602 (32.6)	616 (32.7)
Arthralgia	594 (31.5)	574 (30.7)	601 (32.5)	580 (30.8)
Pain	568 (30.1)	559 (29.9)	588 (31.8)	592 (31.4)
Flu syndrome	489 (25.9)	481 (25.7)	512 (27.7)	509 (27.0)
Infection	462 (24.5)	433 (23.1)	439 (23.7)	452 (24.0)
Abdominal pain	409 (21.7)	413 (22.1)	448 (24.2)	456 (24.2)
Accidental injury^a^	449 (23.8)	394 (21.0)	387 (20.9)	468 (24.8)
Headache	432 (22.9)	446 (23.8)	417 (22.6)	443 (23.5)
Hypertension	396 (21.0)	379 (20.2)	375 (20.3)	394 (20.9)
Constipation	352 (18.7)	356 (19.0)	340 (18.4)	327 (17.3)
Hot flushes^b^	238 (12.6)	244 (13.0)	223 (12.1)	119 (6.3)
Cough increased	216 (11.5)	192 (10.3)	178 (9.6)	199 (10.6)
Leg cramps^a^	207 (11.0)	205 (11.0)	221 (12.0)	156 (8.3)
Asthenia	209 (11.1)	198 (10.6)	212 (11.5)	202 (10.7)
Dyspepsia	192 (10.2)	169 (9.0)	178 (9.6)	195 (10.3)
Peripheral edema	200 (10.6)	184 (9.8)	194 (10.5)	166 (8.8)
Urinary tract infection	183 (9.7)	176 (9.4)	197 (10.7)	170 (9.0)
Dizziness	193 (10.2)	171 (9.1)	165 (8.9)	187 (9.9)
Diarrhea^c^	165 (8.7)	209 (11.2)	211 (11.4)	182 (9.7)

AE, adverse event.

^a^*P *< 0.01; Chi-square test.

^b^*P *< 0.001; Chi-square test.

^c^*P *< 0.05; Chi-square test.

The most frequently reported AEs (≥20% of subjects in at least 1 treatment group) included back pain, arthralgia, pain, flu syndrome, infection, abdominal pain, accidental injury, headache, and hypertension (Table 2). The incidence of leg cramps and hot flushes was higher (overall *P *= 0.002 and *P *< 0.001, respectively) in the active treatment groups compared with the placebo group. Most reports of leg cramps and hot flushes were mild or moderate in severity and did not result in study discontinuation.

### Cardiac disorders

Overall, the number of subjects reporting cardiac disorders (n = 66 [0.9%]) was low and similar among treatment groups. The number of subjects among the bazedoxifene 20- and 40-mg, raloxifene 60-mg, and placebo groups reporting coronary occlusion (2 [0.1%], 0, 0, and 2 [0.1%], respectively), myocardial infarction (8 [0.4%], 8 [0.4%], 6 [0.3%], and 8 [0.4%], respectively), and myocardial ischemia (7 [0.4%], 9 [0.5%], 11 [0.6%], and 9 [0.5%], respectively) was not significantly different.

### Cerebrovascular events

Analysis of the incidence of cerebrovascular events based on adjudicated data showed no significant differences among treatment groups (Table 3). The rate of total strokes per 1,000 women-years was 2.6 with bazedoxifene 20 mg, 3.1 with bazedoxifene 40 mg, 2.6 with raloxifene 60 mg, and 3.0 with placebo (Table 3); corresponding HRs (95% CIs) relative to placebo were 0.9 (0.40, 1.86), 1.0 (0.49, 2.17), and 0.9 (0.40, 1.88), respectively (Table 4). There were no significant differences in the risk of stroke (hemorrhagic, ischemic, or unspecified) or of TIA for bazedoxifene 20 or 40 mg or raloxifene 60 mg compared with placebo (Table 3). There were no deaths due to ischemic stroke in the bazedoxifene or raloxifene treatment groups; there was 1 death due to ischemic stroke in the placebo group. There were 4 deaths due to hemorrhagic stroke (1 in each treatment arm).

**Table 3:** Incidence and Rate per 1,000 Women-Years (95% CI) of Cerebrovascular Events (Adjudicated Data)

	Bazedoxifene		Raloxifene	Placebo
	20 mg (n = 1886)	40 mg (n = 1872)	60 mg (n = 1849)	(n = 1885)
Total stroke				
*n*	12	14	12	14
*Rate*	2.6 (1.34, 4.52)	3.1 (1.70, 5.23)	2.6 (1.36, 4.59)	3.0 (1.65, 5.06)
Hemorrhagic stroke				
*n*	1	1	1	2
*Rate*	0.2 (0.01, 1.20)	0.2 (0.01, 1.24)	0.2 (0.01, 1.22)	0.4 (0.05, 1.55)
Ischemic stroke				
*n*	9	12	10	10
*Rate*	1.9 (0.89, 3.69)	2.7 (1.38, 4.66)	2.2 (1.05, 4.02)	2.2 (1.03, 3.96)
Stroke (unspecified)				
*n*	2	1	1	2
*Rate*	0.4 (0.05, 1.56)	0.2 (0.01, 1.24)	0.2 (0.01, 1.22)	0.4 (0.05, 1.55)
TIA				
*n*	5	7	3	4
*Rate*	1.1 (0.35, 2.52)	1.6 (0.63, 3.21)	0.7 (0.14, 1.92)	0.9 (0.23, 2.20)
Fatal stroke				
*n*	1	1	1	2

CI, confidence interval; TIA, transient ischemic attack.

**Table 4:** HR (95% CI) Versus Placebo for Cerebrovascular Events and VTEs (Adjudicated Data)

	Bazedoxifene		Raloxifene
	20 mg (n = 1886)	40 mg (n = 1872)	60 mg (n = 1849)
Total stroke	0.9 (0.40, 1.86)	1.0 (0.49, 2.17)	0.9 (0.40, 1.88)
Hemorrhagic stroke	0.5 (0.05, 5.53)	0.5 (0.05, 5.70)	0.5 (0.05, 5.65)
Ischemic stroke	0.9 (0.37, 2.22)	1.2 (0.54, 2.87)	1.0 (0.42, 2.44)
Stroke (unspecified)	1.0 (0.14, 7.10)	0.5 (0.05, 5.74)	0.5 (0.05, 5.54)
TIA	1.3 (0.34, 4.68)	1.8 (0.53, 6.18)	0.8 (0.17, 3.41)
Any VTE	1.6 (0.68, 3.94)	1.7 (0.70, 4.07)	1.1 (0.44, 2.96)
DVT	8.0 (1.01, 64.25)	9.4 (1.18, 73.82)	7.1 (0.88, 57.95)
PE	0.8 (0.17, 3.36)	0.8 (0.17, 3.47)	1.0 (0.25, 4.08)
RVT	0.7 (0.11, 4.00)	0.4 (0.04, 3.32)	0
Superficial thrombophlebitis	1.4 (0.55, 3.43)	2.3 (1.01, 5.33)	1.7 (0.68, 3.99)

HR, hazard ratio; CI, confidence interval; VTE, venous thromboembolic event; TIA, transient ischemic attack; DVT, deep vein thrombosis; PE, pulmonary embolus; RVT, retinal vein thrombosis.

### VTEs

A total of 43 subjects (0.6%) reported VTEs based on adjudicated data (Table 5); the overall incidence of VTEs (deep vein thrombosis [DVT], pulmonary embolism [PE], or retinal vein thrombosis [RVT]) was generally higher among the active treatment groups compared with the placebo group. The rates of any VTE per 1,000 women-years were 2.8 with bazedoxifene 20 mg, 2.9 with bazedoxifene 40 mg, 2.0 with raloxifene 60 mg, and 1.7 with placebo (Table 5); corresponding HRs (95% CIs) relative to placebo were 1.6 (0.68, 3.94), 1.7 (0.70, 4.07), and 1.1 (0.44, 2.96), respectively (Table 4). There were no significant differences in the risk of any VTE, including DVT, PE, and RVT, between the bazedoxifene and raloxifene treatment groups. Reports of VTEs were highest during the first year of therapy with bazedoxifene 20 mg and subsequently decreased over the second and third years; no clear pattern over time was observed with bazedoxifene 40 mg or raloxifene 60 mg (Table 5).

**Table 5:** Incidence and Rate per 1,000 Women-Years (95% CI) of VTEs (Adjudicated Data)

	Bazedoxifene		Raloxifene	Placebo
	20 mg (n = 1886)	40 mg (n = 1872)	60 mg (n = 1849)	(n = 1885)
Any VTE				
*n*	13	13	9	8
*Year 1*	8	4	2	3
*Year 2*	4	6	4	2
*Year 3*	1	3	3	3
*Rate*	2.8 (1.49, 4.79)	2.9 (1.54, 4.94)	2.0 (0.90, 3.74)	1.7 (0.74, 3.39)
*Year 1*	4.6 (2.00, 9.14)	2.4 (0.65, 6.09)	1.2 (0.14, 4.29)	1.7 (0.36, 5.05)
*Year 2*	2.7 (0.73, 6.83)	4.2 (1.53, 9.05)	2.7 (0.74, 6.93)	1.3 (0.16, 4.82)
*Year 3*	0.7 (0.02, 3.94)	2.2 (0.45, 6.38)	2.1 (0.44, 6.22)	2.1 (0.44, 6.20)
DVT				
*n*	8	9	7	1
*Rate*	1.7 (0.74, 3.40)	2.0 (0.92, 3.80)	1.5 (0.62, 3.15)	0.2 (0.01, 1.20)
PE				
*n*	3	3	4	4
*Rate*	0.7 (0.13, 1.89)	0.7 (0.14, 1.95)	0.9 (0.24, 2.24)	0.9 (0.23, 2.20)
RVT				
*n*	2	1	0	3
*Rate*	0.4 (0.05, 1.56)	0.2 (0.01, 1.24)	0 (0.00, 0.66)	0.7 (0.13, 1.89)
Superficial thrombophlebitis				
*n*	11	18	13	8
*Rate*	2.4 (1.19, 4.25)	4.0 (2.38, 6.34)	2.9 (1.52, 4.87)	1.7 (0.74, 3.40)

CI, confidence interval; VTE, venous thromboembolic event; DVT, deep vein thrombosis; PE, pulmonary embolism; RVT, retinal vein thrombosis.

The overall incidence of DVTs (n = 25 [0.3%]) and superficial thrombophlebitis (n = 50 [0.7%]) was higher among the active treatment groups compared with the placebo group (Table 5). The rates of DVT per 1,000 women-years were 1.7 with bazedoxifene 20 mg, 2.0 with bazedoxifene 40 mg, 1.5 with raloxifene 60 mg, and 0.2 with placebo (Table 5); corresponding HRs (95% CI) relative to placebo were 8.0 (1.01, 64.25), 9.4 (1.18, 73.82), and 7.1 (0.88, 57.95), respectively (Table 4). There were no differences in the risk of PE or RVT with bazedoxifene 20 or 40 mg or raloxifene 60 mg compared with placebo.

### Breast and reproductive safety

The rates of breast- and reproductive system-related AEs are shown in Table 6. The incidence of breast carcinoma and breast cysts was not significantly different among treatment groups, although the number of cases was lower with bazedoxifene 20 and 40 mg compared with placebo or raloxifene 60 mg. Reports of breast pain were evenly distributed among treatment groups. The incidence of fibrocystic breast disease was lower in the bazedoxifene 20- and 40-mg groups compared with the raloxifene 60-mg or placebo group, and was significantly lower in the bazedoxifene 20-mg group compared with the raloxifene 60-mg group (*P *= 0.050) and in the bazedoxifene 40-mg group compared with the raloxifene 60-mg group (*P *= 0.011).

**Table 6:** Incidence of Breast- and Reproductive System-related AEs

	Bazedoxifene		Raloxifene	Placebo
Subjects, n (%)	20 mg (n = 1886)	40 mg (n = 1872)	60 mg (n = 1849)	(n = 1885)
Breast carcinoma	6 (0.3)	4 (0.2)	7 (0.4)	8 (0.4)
Breast cyst	8 (0.4)	10 (0.5)	17 (0.9)	11 (0.6)
Fibrocystic breast disease^a^	6 (0.3)	4 (0.2)	15 (0.8)	9 (0.5)
Breast neoplasm^b^	12 (0.6)	14 (0.7)	11 (0.6)	22 (1.2)
Breast pain	53 (2.8)	45 (2.4)	56 (3.0)	48 (2.5)
Endometrial carcinoma	0	2 (0.1)	2 (0.1)	3 (0.2)
Endometrial hyperplasia	1 (0.1)	1 (0.1)	1 (0.1)	1 (0.1)
Endometrial neoplasia^c^	9 (0.5)	12 (0.6)	12 (0.6)	10 (0.5)
Ovarian carcinoma	3 (0.2)	0	2 (0.1)	0
Ovarian cyst	16 (0.8)	8 (0.4)	13 (0.7)	14 (0.7)
Uterine hemorrhage	3 (0.2)	5 (0.3)	4 (0.2)	3 (0.2)
Vaginal hemorrhage	16 (0.8)	18 (1.0)	22 (1.2)	23 (1.2)

AE, adverse event.

^a^*P *< 0.05; Chi-square test.

^b^Events reported as breast neoplasm included breast mass, breast lump, solid formation, lipoma, fibroadenoma, tumor, nodule, microcalcification, intracanalar papilloma, and cyst.

^c^Events reported as endometrial neoplasia included endometrial polyps, uterine polyps, thickening of endometrium due to polyps, hyperplastic endometrial polyps, accentuated cystocele and polyps, and endometrial polyps with cystic atrophy (benign).

TVU data were available for a total of 753 subjects; after 2 years, there were no differences in the change in mean endometrial thickness (± standard error) from baseline with bazedoxifene 20 mg (-0.07 ± 0.11 mm), bazedoxifene 40 mg (0.10 ± 0.11 mm), raloxifene 60 mg (0.16 ± 0.12 mm), or placebo (-0.08 ± 0.11 mm). Endometrial hyperplasia was reported by 1 subject in each treatment group, and endometrial carcinoma was reported by 0, 2, 2, and 3 subjects in the bazedoxifene 20- and 40-mg, raloxifene 60-mg, and placebo groups, respectively. The incidence of uterine or vaginal bleeding was low and similar among treatment groups.

### Lipid parameters

The median percent changes from baseline in total and LDL cholesterol were significantly reduced in the bazedoxifene and raloxifene treatment groups compared with the placebo group after 36 months (*P *< 0.001; Table 7). The reduction in total and LDL cholesterol was significantly greater (*P *< 0.01) in the raloxifene treatment group than in the bazedoxifene 20- or 40-mg treatment groups. The increase in levels of HDL cholesterol was significantly greater in the active treatment groups compared with the placebo group after 36 months (*P *< 0.001). Triglyceride levels were significantly increased from baseline in all treatment groups (*P *< 0.001); however, there were no differences in the median percent change from baseline among treatment groups.

**Table 7:** Median Percent Changes From Baseline in Selected Lipid Parameters at Month 36^a^

	Bazedoxifene		Raloxifene	Placebo
Parameter	20 mg	40 mg	60 mg	
Total cholesterol				
*No. of pairs*	1220	1201	1225	1248
*Median percent change*	-3.8^b,c,d^	-3.5^b,c,d^	-5.0^b,c^	0.3
LDL cholesterol				
*No. of pairs*	1220	1201	1223	1247
*Median percent change*	-5.4^b,c,d^	-6.6^b,c,d^	-8.5^b,c^	1.6^b^
HDL cholesterol				
*No. of pairs*	1216	1196	1219	1246
*Median percent change*	5.1^b,c^	5.9^b,c^	5.0^b,c^	2.5^b^
LDL/HDL cholesterol				
*No. of pairs*	1213	1191	1215	1244
*Median percent change*	-10.5^b,c,d^	-12.6^b,c^	-12.4^b,c^	-0.6
Triglycerides				
*No. of pairs*	1220	1201	1225	1248
*Median percent change*	8.5^b^	13.6^b^	12.2^b^	12.1^b^

LDL, low-density lipoprotein; HDL, high-density lipoprotein.

^a^Includes subjects in the intent-to-treat population (n = 6,847) with an assessment at baseline and at Month 36.

^b^*P *< 0.001 vs baseline; t test.

^c^*P *< 0.001 vs placebo; t test.

^d^*P *< 0.01 vs raloxifene 60 mg; t test.

## Discussion

SERMs represent a growing class of compounds that are under development for the prevention and/or treatment of postmenopausal osteoporosis. Because certain "class effects" are associated with SERMs, including increased incidence of hot flushes, leg cramps, and VTEs [11-14], as well as the potential for endometrial effects/stimulation, a careful evaluation of the safety and tolerability profile of each SERM is of considerable importance.

Findings from this large, prospective phase 3 study showed that bazedoxifene was associated with a favorable safety and tolerability profile in postmenopausal women with osteoporosis over 3 years of therapy. Overall, the incidence of AEs, serious AEs, and discontinuations due to AEs with bazedoxifene was similar to that seen with placebo. Although the current study showed an increased incidence of hot flushes and leg cramps in the active treatment groups, most of these events were mild or moderate in severity and did not lead to study discontinuation.

Overall, VTEs were more frequently reported in the bazedoxifene groups compared with the placebo group. This was primarily the result of a significantly increased incidence of DVTs, while the incidence of PE or RVT was not different among all treatment groups. The relative risk of VTEs with bazedoxifene (HR, 1.6-1.7) was consistent with that previously reported with raloxifene (HR, 2.1-3.1) [13,14] and lasofoxifene (HR, 2.2-2.6) [15]. In addition, the rate of VTEs with placebo (1.7 per 1,000 women-years) in this study was similar to that seen in earlier studies of raloxifene (1.7 per 1,000 women-years) [14] and lasofoxifene (1.2 per 1,000 women-years) [15]. Collectively, these findings provide strong external validation of the risk estimates observed with bazedoxifene in the present study.

The rate of VTEs was highest during the first year of therapy with bazedoxifene 20 mg and subsequently decreased over the second and third years; such a pattern was not seen with bazedoxifene 40 mg or raloxifene 60 mg, although a decrease in VTE risk over time has previously been reported with raloxifene (HR, 6.0, 6.6, 0.9 at Years 1, 2, and 3, respectively) [14]. Overall, the absolute risk of VTEs was low among all treatment groups in the current study, with the attributable risk for bazedoxifene estimated at 1 case per 1,000 women-years (ie, 100 women over 10 years of treatment).

Raloxifene has been associated with a 2- to 3-fold increase in VTE risk over 3 years (1.4-fold increase over 5 years) in previous studies [13,14,16], which was higher than that seen in this study. The reason for this discrepancy is unclear and may be attributed to the small number of VTEs reported in the current study. The risk of DVTs, however, was similarly increased with raloxifene relative to placebo in the current study and in previous studies of raloxifene [13,14,16].

The incidence of cardiac disorders and cerebrovascular events in the active treatment groups was not different from that seen in the placebo group. Raloxifene has previously been associated with a small but significant increase in the risk of fatal stroke compared with placebo (1.49; 95% CI, 1.00-2.24; *P *= 0.05), although there was no difference in the overall incidence of stroke [16]. In the current study, there were no deaths due to ischemic stroke with bazedoxifene or raloxifene; there was 1 case of death due to hemorrhagic stroke in each treatment arm.

Endometrial safety is an important consideration for investigational SERMs, as the clinical development of several SERMs for postmenopausal osteoporosis (eg, idoxifene, levormeloxifene) in recent years was discontinued, in part, because of unfavorable uterine effects [17,18]. In this study, bazedoxifene was associated with an excellent endometrial safety profile and a neutral effect on the breast over 3 years of therapy. There was no difference in the rates of endometrial hyperplasia or carcinoma with bazedoxifene compared with placebo, or in the change in endometrial thickness. The incidence of breast- and reproductive system-related AEs were small and similar among groups. There was a lower incidence of fibrocystic breast disease in the bazedoxifene treatment groups compared with the raloxifene or placebo group. Raloxifene was not associated with a reduction in the incidence of breast cancer in this study, which is in contrast to findings from previous studies [16,19,20] and may be attributable to the relatively smaller number of subjects evaluated and the lower baseline risk for breast cancer (mean Gail Index 5-year risk score of 1.59). In this study, the overall incidence of breast cancer in the placebo groups was lower than that observed in other clinical trials [8,14,19].

Bazedoxifene was associated with favorable effects on the lipid profile. Significant reductions in the levels of total and LDL cholesterol and significant increases in levels of HDL cholesterol were observed with bazedoxifene compared with placebo. Triglyceride levels were similar among all treatment groups. The clinical significance of these results is unknown. Overall, findings are consistent with those seen in the 2-year phase 3 study evaluating the efficacy and safety of bazedoxifene in postmenopausal women at risk for osteoporosis [7].

## Conclusions

Overall, bazedoxifene was associated with a favorable safety and tolerability profile over 3 years of therapy, with no evidence of endometrial or breast stimulation. Bazedoxifene was associated with a small increase in the risk of VTEs, consistent with that seen with other SERMs, including raloxifene and lasofoxifene; however, the absolute risk of VTEs was low and did not increase over time. Combined with its demonstrated efficacy in preventing new vertebral fractures in postmenopausal women with osteoporosis, as well as in preventing nonvertebral fractures in higher-risk women [9], bazedoxifene appears to have a favorable risk-benefit profile in the treatment of postmenopausal women with osteoporosis.

## Competing interests

Dr Christiansen has served as a consultant for Wyeth, Eli Lilly, Roche, Novartis, Novo Nordisk, Procter & Gamble, Groupe Fournier, Besins EscoVesco, MSD, Chiesi, Boehringer Mannheim, and Pfizer. Dr Chesnut has been a consultant and speaker for Amgen. Dr Adachi has been a consultant and speaker for Amgen, AstraZeneca, Bristol-Myers Squibb, Eli Lilly, GlaxoSmithKline, Merck, Novartis, Nycomed, Pfizer, Procter & Gamble, Roche, sanofi-aventis, Servier, and Wyeth. Dr Brown has been an investigator for Wyeth and has served as a consultant for and/or received honoraria or research funding from Abbott, Amgen, ArthroLab, Bristol-Myers Squibb, Eli Lilly, GlaxoSmithKline, Merck Frosst, Novartis, Nycomed, Pfizer, Procter & Gamble, Roche, sanofi-aventis, Servier, and Wyeth. Dr Fernandes has been an investigator for Wyeth and Eli Lilly. Dr Kung has received research support and speakers honoraria from Eli Lilly, MSD Asia Pacific, Novartis, Roche, and Servier. Dr Palacios has been a symposium speaker or advisory board member for Bayer Schering Pharma, Novo Nordisk, Servier, Lilly, Daiichi-Sankyo, sanofi-aventis, MSD, and Procter & Gamble. He has also received research grants and/or consulting fees from Wyeth, Servier, Lilly, Daiichi-Sankyo, Amgen, Arkochim, and Bayer Schering Pharma. Drs Levine, Chines, and Constantine were previous employees of Wyeth, which was acquired by Pfizer Inc in October 2009.

## Authors' contributions

CC, CHC, JDA, JPB, CEF, AWCK, SP, and ABL analyzed and interpreted data; AAC participated in the acquisition of data and analyzed and interpreted data; GDC was responsible for the conception and design of the trial and analyzed and interpreted data. All authors critically revised the manuscript for important intellectual content and read and approved the final manuscript.

## Pre-publication history

The pre-publication history for this paper can be accessed here:

http://www.biomedcentral.com/1471-2474/11/130/prepub
